# Nuclear factor kappa B modulates apoptosis in the brain endothelial cells and intravascular leukocytes of fatal cerebral malaria

**DOI:** 10.1186/1475-2875-12-260

**Published:** 2013-07-26

**Authors:** Chuchard Punsawad, Yaowapa Maneerat, Urai Chaisri, Kwannan Nantavisai, Parnpen Viriyavejakul

**Affiliations:** 1 Department of Tropical Pathology, Faculty of Tropical Medicine, Mahidol University, 420/6 Rajvithi Road, Bangkok 10400, Thailand; 2 School of Medicine, Walailak University, 222 Thasala District, Nakhon Si Thammarat 80161, Thailand; 3 Department of Microbiology, Faculty of Medicine, Srinakarinwiroj University, 114 Sukhumvit 23, Bangkok 10110, Thailand; 4 Center for Emerging and Neglected Infectious Diseases, Mahidol University, Bangkok 10400, Thailand

**Keywords:** Malaria, Cerebral malaria, Nuclear factor kappa B, NF-κB p65, Cleaved caspase-3, Apoptosis, Immunohistochemistry

## Abstract

**Background:**

Cerebral malaria (CM) caused by *Plasmodium falciparum* is known to be associated with the sequestration of parasitized red blood cells (PRBCs) in the microvasculature and the release of soluble cytokines. In addition, the involvement of signaling molecules has gained wide interest in the pathogenesis of CM. An important signaling factor, nuclear factor kappa B (NF-κB) is known to regulate apoptosis. This work aimed to study the expression of NF-κB p65 and its correlation with apoptosis in the brain of fatal CM.

**Methods:**

The expression of NF-κB p65 and cleaved caspase-3 in the brain of fatal *P. falciparum* malaria cases was investigated by immunohistochemistry. Histopathological features were analysed together with the correlations of NF-κB p65 and cleaved caspase-3 expression.

**Results:**

NF-κB p65 activation and cleaved caspase-3 expression were significantly increased in the neurons, glial cells, vascular endothelial cells (ECs) and intravascular leukocytes of the brain in fatal CM, compared with the control brain (*p* < 0.001) and non-cerebral malaria (NCM) (*p* = 0.034). The percentage of neurons that expressed nuclear NF-κB p65 showed a positive correlation with the total score of histopathological changes (*r*_*s*_ = 0.678; *p* = 0.045). Significant positive correlations were established between vascular ECs NF-κB index and ECs apoptotic index (*r*_*s*_ = 0.717; *p* = 0.030) and between intravascular leukocytes NF-κB index and leukocytes apoptotic index (*r*_*s*_ = 0.696; *p* = 0.037) in fatal CM.

**Conclusions:**

This study documented that NF-κB p65 is one of the signaling factors that modulates apoptosis in the brain ECs and intravascular leukocytes of fatal CM.

## Background

The process of malaria pathogenesis is very complex. Despite malaria being one of the most extensively studied infectious diseases over the past decade, the exact molecular basis for disease progression remains poorly understood. However, several key processes can be recognized; these include the adherence of parasitized red blood cells (PRBCs) to microvascular endothelial cells, leading to vascular obstruction and subsequent cerebral hypoxia [1] and the production of high levels of pro-inflammatory mediators during acute malaria infection [2]. The attachment of PRBCs is mediated by specific host endothelial receptor molecules, predominantly intercellular adhesion molecules 1 (ICAM-1) in the brain [3]. Several investigations have shown that the expression of adhesion molecules on the endothelium is closely linked to the activation of transcription nuclear factor kappa B (NF-κB) protein [4-6]. Recently, Tripathi *et al.* showed that PRBC exposure to human brain endothelium can induce a predominantly pro-inflammatory response that is mediated by activation of NF-κB [4]. NF-κB is a transcription factor that regulates the expression of various genes involved in cell proliferation, adhesion, anti-apoptosis and apoptosis [5]. One of the important events regulated by NF-κB is apoptosis, a complex biological process of cell death. This process can be mediated by various stimuli, including hormones, cytokines, growth factors, bacterial or viral infections and immune response [6]. An important and reliable indication of apoptosis induction is the detection of cells that express the active form of caspase-3 [7], an executor enzyme which can be activated by several other active caspases. *In vitro* evidence further suggests that PRBCs, leukocytes, and platelets are directly involved in endothelial cell damage and might, therefore, contribute to blood–brain barrier (BBB) disruption by apoptotic cell death [8-10]. In addition, soluble cytokines such as tumor necrosis factor (TNF) are known to alter BBB permeability [11], allowing cytokines and malaria antigens to enter the brain compartment. These could lead to the activation of the microglia or damage to glial cells, which have been observed in murine and human cerebral malaria [12-15]. Recently, axonal injury was demonstrated in the brain of cerebral malaria (CM) patients [16]. However, the underlying mechanisms of neuronal and glial degeneration are not yet sufficiently understood. Although it is known that increased production of pro-inflammatory cytokines is an important process that contributes to the pathogenesis of *Plasmodium falciparum* malaria, very little is known about the nature of the various potential ligands of the parasite and the signaling mechanisms involved.

In this study, the involvement of NF-κB p65 activation and caspase-3 expression was investigated by immunohistochemistry in postmortem brain tissues of patients who died from *P. falciparum* malaria. The correlation between NF-κB p65 and caspase-3 expression was analysed with the clinical parameters and histopathological changes seen in fatal CM.

## Methods

### Brain tissue specimens

The paraffin-embedded brain tissues from cerebral malaria (CM) patients and non-cerebral malaria (NCM) patients at the Department of Tropical Pathology, Faculty of Tropical Medicine, Mahidol University, Thailand, from 1995 to 2004, were enrolled into this study. Normal brain tissues (n = 10) were obtained from patients who had died from accidents without head injury. The use of left-over brain specimens and the study protocol were reviewed and approved by the Ethics Committee of the Faculty of Tropical Medicine, Mahidol University (MUTM 2010-053-01, MUTM 2010-053-02).

### Histopathological examination

Brain sections were stained with modified haematoxylin and eosin (H&E) stain, according to routine methods. Representative brain tissues were randomly obtained from cerebellum (frontal, temporal and occipital lobes) and cerebellum. Three parameters of histopathological features in the brain section were evaluated- including vascular sequestration with PRBCs, ring haemorrhage and Durck’s granuloma. For the semi-quantitative analysis of histopathological changes, a grading scale (range from 0 to 10 points) was developed to determine CM-related histopathological changes (Table 1). A score of 0 meant the absence of PRBC sequestration, ring haemorrhage and Durck’s granuloma, while a score of 10 signified the most severe histopathological changes.

**Table 1:** Three features, 10-points histological scoring devised to determine histopathological changes in the brain of fatal malaria cases

**Parameters**	**Histological characterization**	**Score**
Sequestration of PRBCs	None	0
	≤ 30%/HPF	1
	31-50%/HPF	2
	51-70%/HPF	3
	71-90%/HPF	4
	> 91%/HPF	5
Ring haemorrhage	None	0
	≤ 10 areas/slide	1
	11-20 areas/slide	2
	21-30 areas/slide	3
Durck’s granuloma	None	0
	≤ 2 areas/slide	1
	> 2 areas/slide	2
**Total Score: (5) + (3) + (2) = 10**		

*HPF* = high power field (X400).

### Immunohistochemistry for NF-κB p65 and cleaved caspase-3

Brain tissues were sectioned at 4 μm in thickness, deparaffinized in xylene and rehydrated through graded concentrations of alcohol. Subsequently, antigen was retrieved in 0.1 M Tris–HCl buffer (pH 9.0) for NF-κB p65 and 0.1 M sodium citrate buffer (pH 6.0) for cleaved caspase-3 by microwave method. The endogenous peroxidase was inactivated with 3% hydrogen peroxide in distilled water for 30 min at room temperature. After washing the sections in phosphate-buffered saline (PBS, pH 7.4), the nonspecific binding site was blocked with 10% normal goat serum for 30 min at room temperature. Sections were incubated overnight at 4°C with specific primary antibody; mouse monoclonal antibody directed against the p65 (F-6) RelA component of the NF-κB complex (1:50; Santa Cruz Biotechnology, CA, USA), and rabbit polyclonal antibody that recognized the large fragment (17/19 kDa) of cleaved caspase-3 (1:200; Cell Signaling Technology, USA). During the ensuing days, sections were washed three times with PBS and incubated with secondary antibody for 30 min at room temperature and reacted with avidin-biotin complex (ABC) conjugated with horseradish peroxidase (HRP) (for NF-κB p65) or alkaline phosphatase (AP) (for cleaved caspase-3) (Vectastain ABC kit, Vector Laboratories, CA, USA), according to the manufacturer’s instructions. After washing with PBS, enzymatic activity was visualized by 3,3'-diaminobenzidine tetrahydrochloride (DAB) or Vector® Red substrate kit (Vector Laboratories, CA, USA) for peroxidase (brown color) and alkaline phosphatase (red color) activity, respectively. Sections were counterstained with haematoxylin, then dehydrated and mounted with a coverslip.

### Positive and negative controls

Known positive controls (breast cancer for NF-κB p65 and lymph node for cleaved caspase-3) were used to confirm the specificity of primary antibodies and validate the immunohistochemistry staining techniques. The negative control was implemented by omission of primary antibody, with staining in each run. In addition, brain sections were incubated with the immunohistochemistry substrate, DAB or Vector® Red substrate, to confirm the presence of endogenous peroxidase and alkaline phosphatase activity.

### Evaluation of NF-κB p65 and cleaved caspase-3 immunostaining

The quantitative immunoreactivity of NF-κB p65 was evaluated in nuclear NF-κB p65 stained cells. Cleaved caspase-3 stained with red color in the cytoplasm and/or nucleus of a cell was considered an apoptotic cell. The expressions of NF-κB p65 and cleaved caspase-3 immunostaining were examined in neurons, glial cells, endothelial cells (ECs) and intravascular leukocytes. In each case, five brain sections (three from cerebrum and two from cerebellum) were evaluated. Each immunostained section was randomly counted in 10 microscopic fields under high-power field (X400) microscope. In each field, positive cells and total cell number were recorded and added up with all 50 microscopic fields then percentage of positive stained cells (%) was calculated as the number of positive cells divided by the total cell count, multiplied by 100. The staining intensity was graded on a scale (range 0 to 3) by semi-quantitative assessment, as follows: 0 = negative, 1 = weak staining, 2 = moderate, and 3 = strong. Finally, the total score (TS) was calculated by multiplying percentage of immunopositive cells (P) by staining intensity (I) [Total score (TS) = percentage of positive cells (P) X staining intensity (I); maximum = 300], which was modified from previous studies [17,18]. Nuclear NF-κB and apoptotic indices in ECs and intravascular leukocytes were measured in blood vessels between 10 to 50 μm in diameter. Finally, the index was calculated by dividing the total number of immunopositive cells by the total number of blood vessels. Histopathology and immunohistochemistry were evaluated by two independent observers (CP and PV) unaware of the patients’ clinical outcome. When inter-observer disagreement was noted, a third investigator evaluated the sample.

### Statistical analysis

Results were presented as mean ± standard error of the mean (SEM). The normality of distribution was tested by Kolmogorov-Smirnov test. Differences between groups were analysed by Mann Whitney *U-*test. Spearman’s rank correlation coefficient (*r*_*s*_) was computed to estimate the direction and strength of correlations between the expression of NF-κB p65 and cleaved caspase-3, histopathological changes, and clinical data. Statistical analysis was conducted using SPSS version 17.0 software (SPSS, IL, USA). A *p* value < 0.05 was considered significantly different.

## Results

### Clinical data of fatal CM and NCM cases

Brain-tissue specimens were obtained from nine patients who had died of cerebral *P. falciparum* malaria and two from NCM; their clinical data appear in Table 2. The CM group comprised three women (age range 3 to 35 years) and six men (age range 8 to 41 years). The following clinical features were obtained on admission for the CM patients: the mean haemoglobin concentration was 11.3 ± 0.9 g/dl, mean white blood cell (WBC) count was 18,486 ± 3,947.8 cells/mm^3^, mean time from admission to death was 47 ± 17.5 hr, mean parasite count on admission was 564,605 ± 259,125 parasites/μl, and mean last parasite count was 377,042 ± 395,839 parasites/μl. Other complications associated with CM included anaemia (5/9, 55.6%), jaundice (7/9, 77.8%), pneumonia (5/9, 55.6%), shock (2/9, 22.2%), pulmonary oedema (5/9, 55.6%), disseminated intravascular coagulation (DIC) (2/9, 22.2%), acute kidney injury (AKI) (4/9, 44.4%), hypoglycemia (2/9, 22.22%) and acidosis (1/9, 11.1%). The NCM group comprised two cases. The significant differences in clinical parameters between the CM and NCM groups were mean parasite count on admission (*p* = 0.034) and mean last parasite count (*p* = 0.0033).

**Table 2:** Clinical details of fatal malaria cases

**Code no.**	**Group**	**Age (Year)**	**Sex**	**Hb**^**† **^**(g/dl)**	**WBC**^**† **^**(μl)**	**Time to death (hr)**	**Admission parasite count/μl**	**Last parasite count/μl**	**Other complications**
A001	CM	3	F	12	ND	12	677,040	677,040	Anaemia, Jaundice, Pneumonia, Shock, PE
A002	CM	18	M	8	28,200	4	29,900	29,900	Anaemia, Jaundice, PE
A003	CM	15	M	12	18,350	0.5	190,000	190,000	Jaundice, Pneumonia, DIC, PE
A004	CM	28	M	7.3	32,500	96	20,460	3,410	Anaemia, Jaundice, Pneumonia, AKI
A005	CM	35	F	14.6	6,941	144	52,780	81,200	AKI
A006	CM	41	M	14.3	23,250	13	521,730	521,730	Pneumonia, Shock
A007	CM	18	M	13.2	4,350	48	2,494,940	702,800	Jaundice, AKI, Hypoglycemia
A008	CM	13	M	10.7	6,600	96	313,900	64,500	Anaemia, Jaundice, Pneumonia, Hypoglycemia, PE
A009	CM	8	F	9.5	27,700	8	780,700	1,122,806	Anaemia, Jaundice, AKI, Acidosis, DIC, PE
A101	NCM	65	M	11.4	9,312	120	150	0	Pneumonia, PE
A102	NCM	38	M	6.8	4,800	144	96	0	Metabolic acidosis, Pneumonia, PE

*F* = female; *M* = male; ^†^data on admission; *CM* = cerebral malaria; *NCM* = non-cerebral malaria; *PE* = pulmonary oedema; *DIC* = disseminated intravascular coagulation; *AKI* = acute kidney injury, *ND* = no data.

### Histopathological observations in the brain of fatal CM and NCM patients

Histopathological changes in the brains of fatal *P. falciparum* malaria cases are summarized in Table 3. The total score for histopathological changes was semi-quantitatively assessed in each specimen. For the CM group, the highest score for PRBC sequestration (Score 5) was observed in 4 cases (44.4%). PRBC sequestration was found in the brain sections of all cases with scores ranging from 1 to 5. The highest score for areas of ring hemorrhage was seen in 2 cases (22.2%). Durck’s granuloma, showing a reactive gliosis resembling the formation of granulomas, was observed in 2 cases (22.2%). The mean total score for overall histopathological changes in the brains of the fatal CM cases was 4.4 points, whereas no significant histopathological changes were observed in the brain sections of the NCM cases.

**Table 3:** Histopathological changes in the brains of fatal CM and NCM patients

**Histopathological changes**					
**Code no.**	**Group**	^**a**^**PRBC sequestration**	^**b**^**Ring haemorrhage**	^**c**^**Durck’s granuloma**	^**†**^**Total score**
A001	CM	5	1	0	**6**
A002	CM	5	3	0	**8**
A003	CM	3	0	0	**3**
A004	CM	1	3	1	**5**
A005	CM	1	1	0	**2**
A006	CM	5	1	0	**6**
A007	CM	5	0	0	**5**
A008	CM	1	0	2	**3**
A009	CM	1	0	1	**2**
A101	NCM	0	0	0	**0**
A102	NCM	0	0	0	**0**

^a^PRBC sequestration range: 0 to 5 points, ^b^Ring haemorrhage range: 0 to 3 points, ^c^Durck’s granuloma range: 0 to 2 points.

^†^Total score range: 0 to 10 points. A score of 0 signifies no abnormal histopathological changes, while a score of 10 signifies the most severe histopathological changes.

### Expression of NF-κB p65 in neurons and glial cells

**Figure 1:**
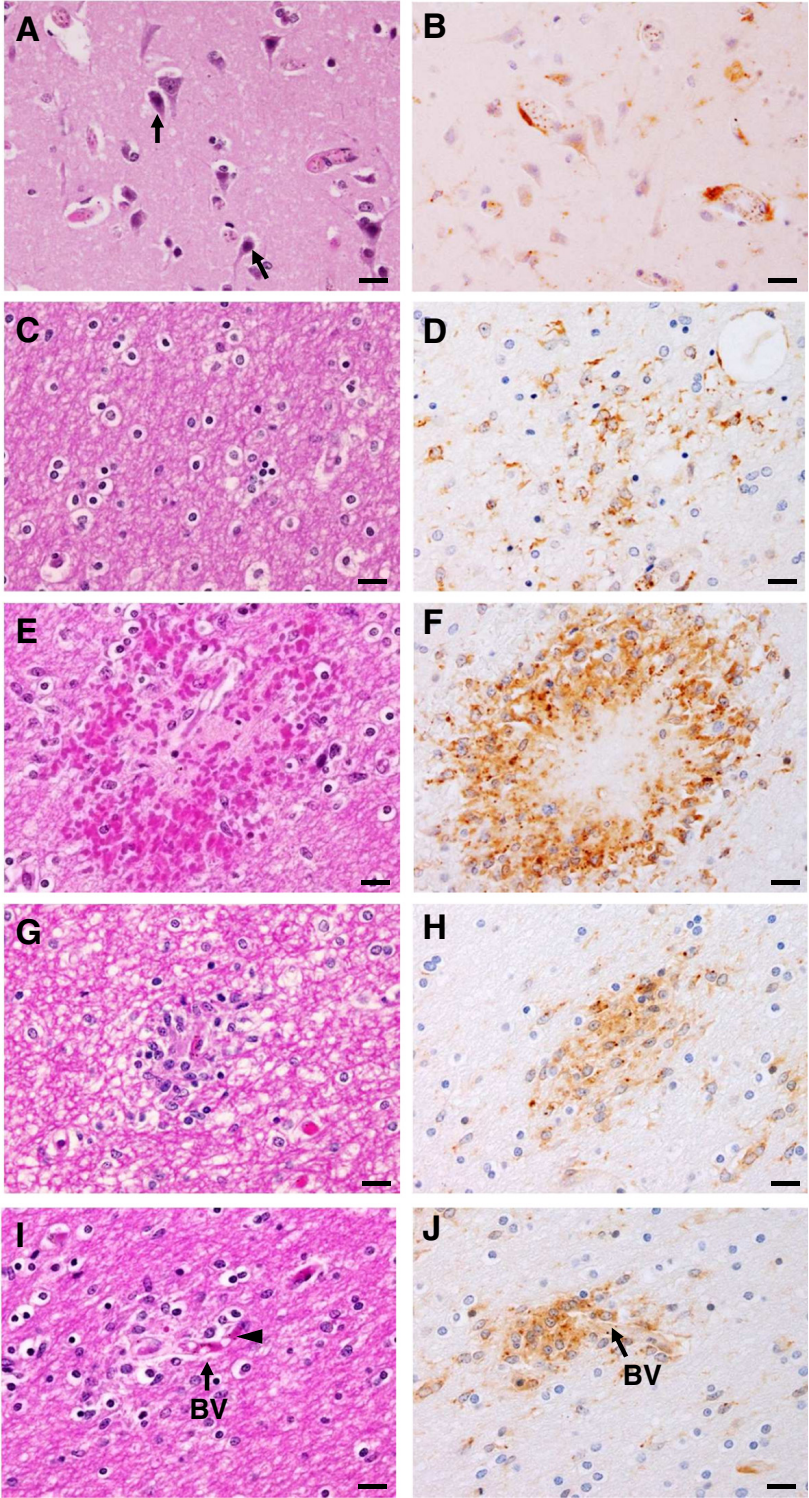
**Histopathologic changes (A, C, E, G, I) and pattern of immunohistochemistry staining for NF-κB p65 (B, D, F, H, J) in the brain of fatal cerebral malaria. (A)** Group of neurons **(H**&**E)**. Apoptotic features of cellular shrinkage, apoptotic nuclei and increased cytoplasmic staining for eosin were observed in some neurons (arrows). **(B)** Neurons demonstrating predominantly cytoplasmic NF-κB p65 staining. **(C)** Glial cells in white matter **(H**&**E)**. **(D)** Group of glial cells showing cytoplasmic and nuclear NF-κB p65 immunostaining **(E)** Ring haemorrhage in white matter **(H**&**E)**. **(F)** Glial cells in area of haemorrhage showed strong positive staining for cytoplasmic and nuclear NF-κB p65. **(G)** Mass of reactive glial cells forming an ill-defined granuloma collectively called Durck’s granuloma **(H**&**E)**. **(H)** Reactive glial cells showed intense positive staining for cytoplasmic and nuclear NF-κB p65. **(I)** Group of glial cells associated with blood vessel (arrow) containing PRBCs (arrowhead) **(H**&**E)**. **(J)** Glial cells stained for nuclear and cytoplasmic NF-κB p65 associated with blood vessel (arrow). Scale bars = 20 μm.

**Figure 2:**
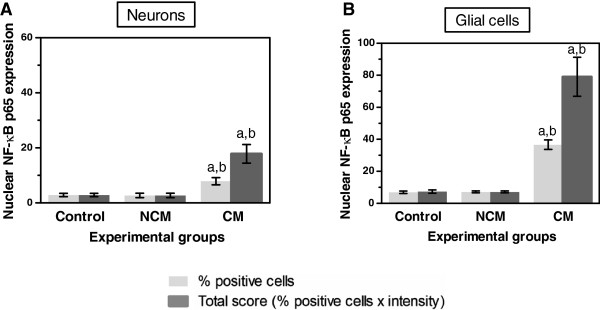
**Expression of nuclear NF-κB p65 in the brain of control, NCM, and CM groups in neurons (A) and glial cells (B). **^a^Significance of *p* < 0.001 compared with control brain (Mann–Whitney U test). ^b^Significance of *p* = 0.034 compared with NCM (Mann–Whitney U test). Data are presented as mean ± SEM.

Localized NF-κB p65 immunostaining was detected in the cytoplasm and/or nucleus of cellular components in the brain--neurons, glial cells, vascular ECs, and intravascular leukocytes. Cytoplasmic NF-κB p65 expression represented an inactive protein state bound to inhibitor kappa B (IκB), while NF-κB expression in the nucleus represented an active state. Since NF-κB p65 activation results in nuclear translocation [19], only nuclear-stained cells were considered and counted for quantitative analysis. Figure 1 represents comparative histopathologic changes stained with modified H&E stain. Figure 1 represents histopathologic changes stained with modified H&E stain (left panel- A, C, E, G, I) and comparative immunostaining pattern for NF-κB p65 (right panel- B, D, F, H, J). Neurons were mostly positive for cytoplasmic NF-κB p65 staining, indicating NF-κB in an inactive state (Figure 1B). However, many glial cells in the white matter were frequently found to express nuclear NF-κB p65 staining (Figure 1D) as well as glial cells located in areas of ring haemorrhage (Figure 1F). Additionally, a collection of activated glial cells called Durck’s granuloma (Figure 1H), and glial cells closely associated with blood vessels (Figure 1J), mostly showed intense nuclear NF-κB p65 expression. Different brain sections from the cerebrum and cerebellum showed similar immunoreactive cells and staining intensity. The mean percentages and total scores for nuclear NF-κB p65 immunostaining in neurons and glial cells are shown in Figure 2. NF-κB p65 expression in the neurons and glial cells in the CM cases was significantly greater than the control brain (all *p* < 0.001) and NCM (all *p* = 0.034). To observe the ratio of the glial cell population expressing nuclear NF-κB p65, glial cell subtypes were identified by cell morphology. Oligodendrocytes had significantly higher mean percentage nuclear NF-κB p65 than astrocytes (*p* < 0.001).

### Expression of NF-κB p65 in vascular ECs and intravascular leukocytes

**Figure 3:**
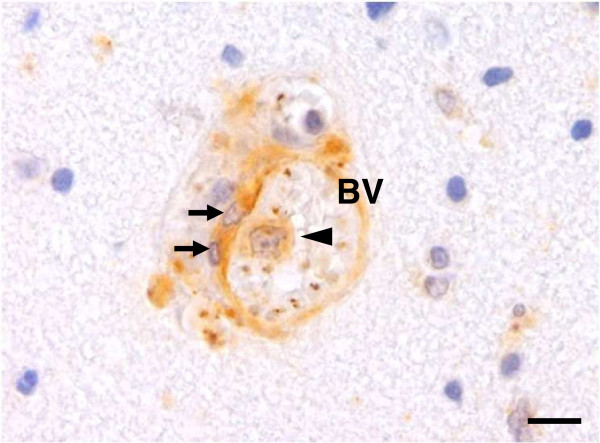
**Immunohistochemical staining of NF-κB p65 in ECs and leukocytes in the brains of CM cases.** This micrograph illustrates a cross-section of a blood vessel with strong immunopositive staining for NF-κB p65 in ECs (arrows) and an adhered leukocyte (arrowhead). BV = blood vessel. Scale bars = 10 μm.

**Figure 4:**
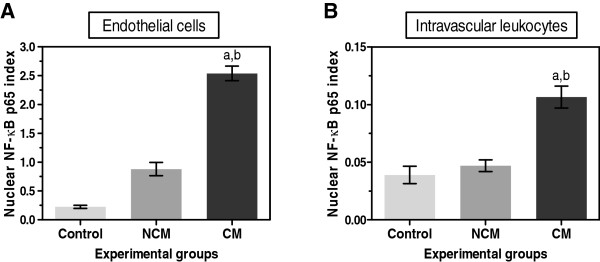
**Quantitative analysis of nuclear NF-κB p65 index in vascular ECs and in intravascular lymphocytes in the brains of control, NCM and CM groups.** The graphs show NF-κB p65 expression in vascular ECs **(A)** and intravascular leukocytes **(B)** in three experimental groups. ^a^Significance of *p* < 0.001 compared with control brain (Mann–Whitney *U* test). ^b^Significance of *p* = 0.034 compared with NCM (Mann–Whitney *U* test). Data are presented as mean ± SEM.

It is known that the PRBC-sequestration and cytokine-mediated mechanism are involved in the pathogenesis of CM. Vascular ECs are an important attachment site and the target of PRBC cytoadhesion. The results demonstrated that vascular ECs show a strong nuclear immunoreactivity against NF-κB p65 in CM (Figure 3). Sixty percent (60.4% ± 5.1) of ECs were immunopositive for NF-κB p65 as compared to 29.7% ± 4.8 in control group (*p* = 0.002) and 30.0% ± 5.0 in NCM group (*p* = 0.044). Circulating inflammatory cells particularly lymphocytes are seen in CM. Intravascular lymphocytes shows higher nuclear immunoreactivity against NF-κB p65 than control and NCM, which indicates the active state of these cells during malaria infection. To better characterize the expression of NF-κB p65 in vascular ECs and intravascular leukocytes, the number of immunopositive cells per blood vessel was counted and calculated for a nuclear NF-κB p65 index. The ECs NF-κB index and intravascular leukocytes NF-κB index were significantly higher in the brains of fatal CM cases than controls (all *p* < 0.001) and NCM (all *p* = 0.034) (Figure 4).

### Expression of cleaved caspase-3 in neurons and glial cells

**Figure 5:**
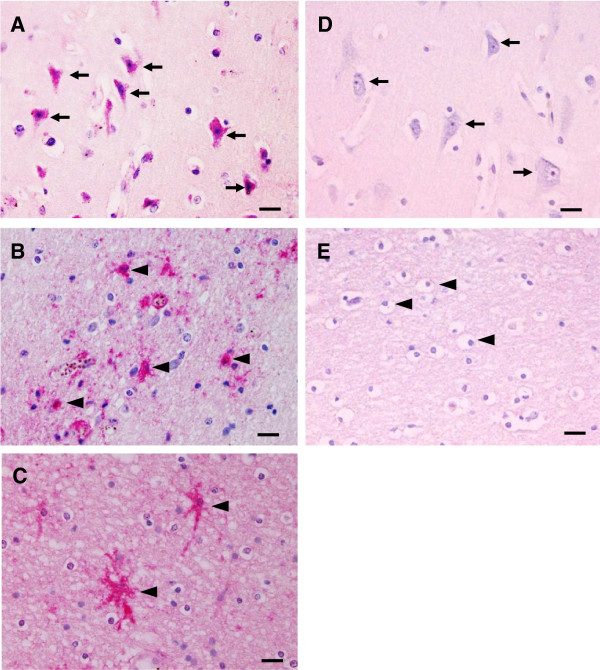
**Pattern of immunohistochemical staining for cleaved caspase-3 in the brains of fatal CM cases.** A strong neuronal immunoreactivity for cleaved caspase-3 was observed in the neurons (arrows) **(A)** and glial cells (arrowheads) **(B and C)** compared with the neurons **(D)** and glial cells **(E)** of the normal brain. Scale bars = 20 μm.

**Figure 6:**
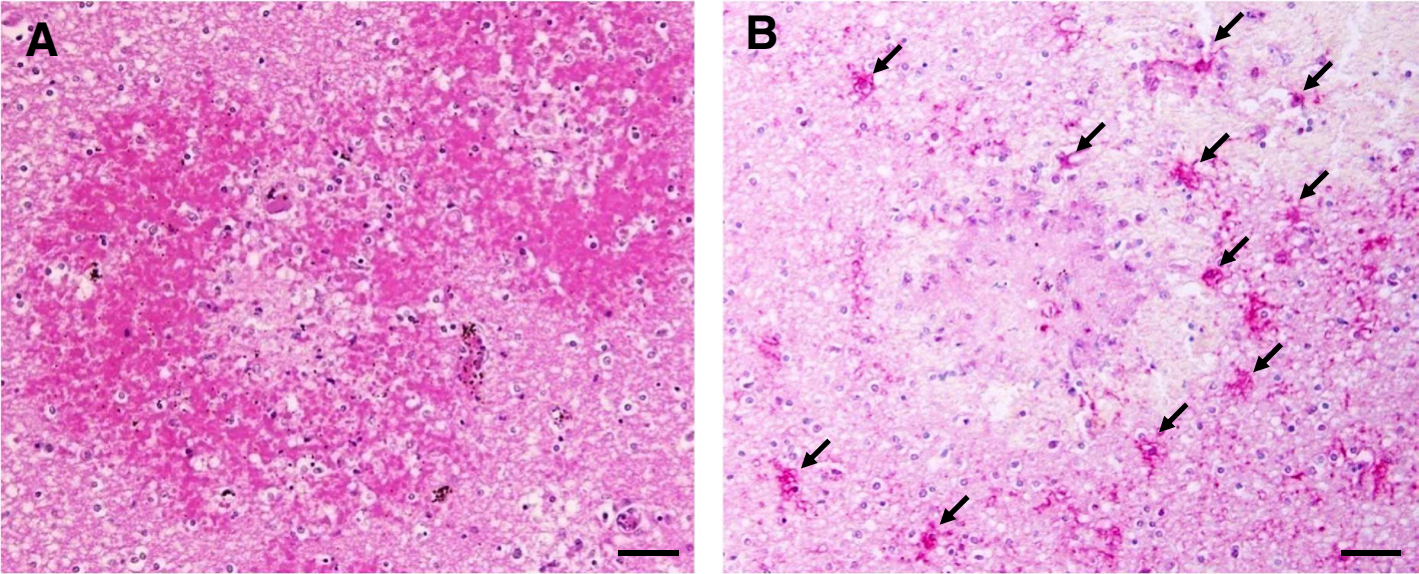
**Immunohistochemical staining for cleaved caspase-3 in the area of haemorrhage in the brains of fatal CM cases. (A)** Representative photograph of area of congestion and ring haemorrhage is shown with H&E stain. **(B)** Many glial cells located around and within the area of haemorrhage show strong positive staining for cleaved caspase-3 (arrows). Scale bars = 50 μm.

**Figure 7:**
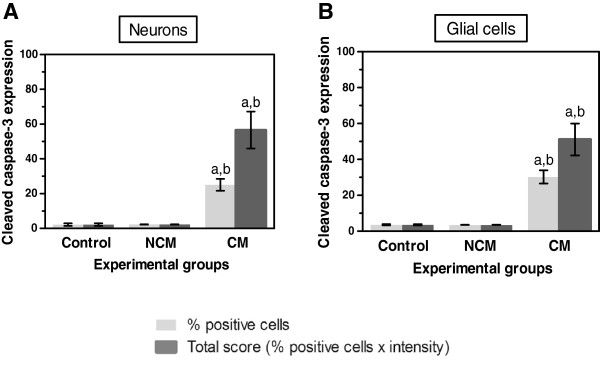
**Quantification of cleaved caspase-3 expression in neurons (A) and glial cells (B) in the brain of the control, NCM, and CM groups. **^a^Significance of *p* < 0.001 compared with control brain (Mann–Whitney *U* test). ^b^Significance of *p* = 0.034 compared with NCM (Mann–Whitney *U* test). Data are presented as mean ± SEM.

One of the important events regulated by NF-κB p65 is apoptosis. To evaluate whether apoptosis detected at the final apoptotic cascade pathway (caspase-3) is involved in the cause of CM, brain sections from fatal CM patients were investigated to determine the expression of cleaved caspase-3. Immunostaining patterns for cleaved caspase-3 showed homogenous staining in both the cytoplasm and nucleus. Localization of cleaved caspase-3 in the brain of fatal CM is shown in Figure 5. Neurons were frequently positive for cleaved caspase-3 (Figure 5A). Glial cells were reactive and showed strong immunostaining (Figure 5B). Glial-cell processes exhibited radial immunostaining of cleaved caspase-3, resembling a spiderweb pattern (Figure 5C). There was no detectable cleaved caspase-3 immunoreactivity in the brains of the control group (Figures 5D and 5E) and to a lesser degree in the NCM group. Interestingly, cleaved caspase-3 in the glial cells was frequently found around and within areas of ring haemorrhages (Figure 6). For quantification, immunoreactivity and the total score for cleaved caspase-3 was significantly higher in neurons and glial cells than in the control (all *p* < 0.001) and NCM (all *p* < 0.001) groups (Figure 7). In addition, oligodendrocytes showed significantly higher cleaved caspase-3 expression than astrocytes in the brains of fatal CM cases (*p* < 0.001).

### Expression of cleaved caspase-3 in vascular ECs and intravascular leukocytes

**Figure 8:**
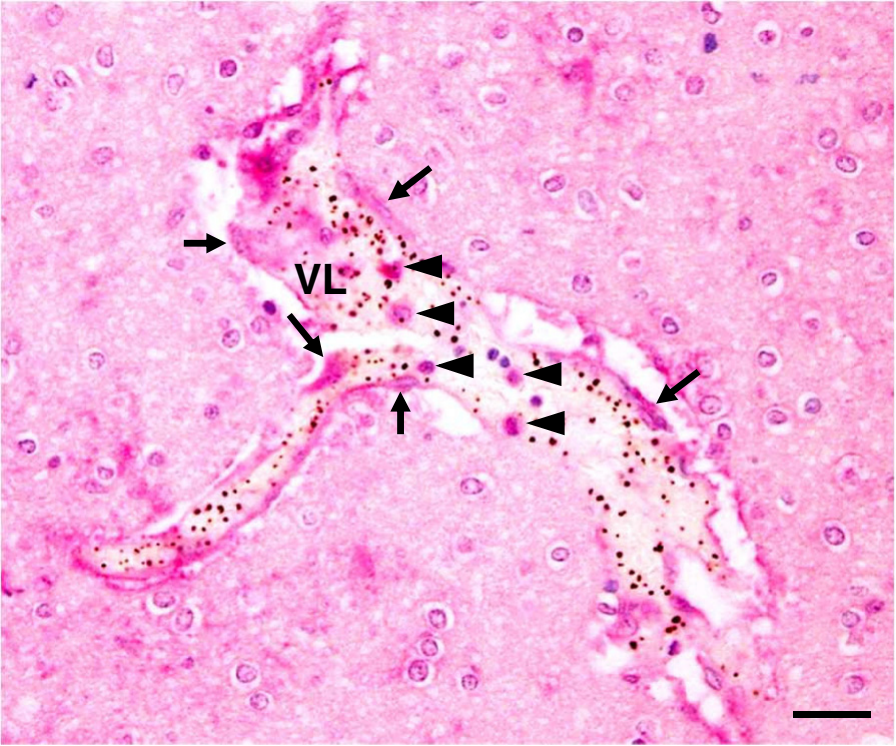
**Immunohistochemical staining for cleaved caspase-3 in the blood vessel of fatal CM brain.** A micrograph to demonstrate strong positive staining for cleaved caspase-3 in the ECs (arrows) sequestered with PRBCs and leukocytes (arrowheads) inside the vascular lumen. VL = vascular lumen. Scale bars = 20 μm.

**Figure 9:**
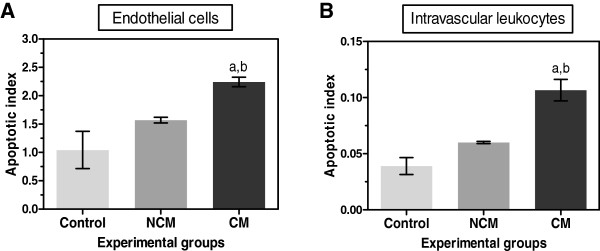
**Quantification of cleaved caspase-3 in vascular ECs and intravascular leukocytes in the brain of control, NCM and CM groups.** Data were analysed as apoptotic index in the vascular ECs **(A)** and intravascular leukocytes **(B)**. ^a^Significance of *p* < 0.001 compared with control brain (Mann–Whitney U test). ^b^Significance of *p* = 0.034 compared with NCM (Mann–Whitney U test). Data are presented as mean ± SEM.

In addition to the parenchymal cells, ECs immunopositive for activated caspase-3 were observed in the blood vessels containing PRBC sequestration as well as both circulating and adherent leukocytes (Figure 8). The percentage of ECs immunopositive for cleaved caspase-3 was significantly increased in CM (77.4% ± 3.8) compared to control (2.0% ± 2.0, *p* = 0.000) and NCM groups (2.5% ± 2.5, *p* = 0.034). To determine the occurrence of apoptosis in the vascular ECs and intravascular leukocytes, the apoptotic index, indicating the number of immunopositive ECs or leukocytes stained for cleaved caspase-3 per blood vessel, was used to measure the degree of apoptosis in ECs and leukocytes. The vascular ECs apoptotic index and the intravascular leukocyte apoptotic index were significantly higher in fatal CM than in the control brain and NCM group (all *p* < 0.001) and NCM group (all *p* = 0.0.034) (Figure 9).

### Correlations of NF-κB p65 and caspase-3 expression with histopathological changes and clinical findings

**Figure 10:**
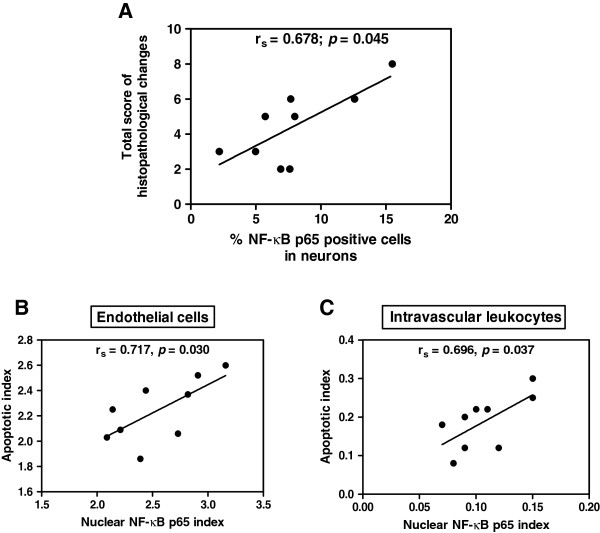
The graphs illustrate positive correlations between total score for histopathological changes and NF-κB p65 (A), apoptotic index and NF-κB p65 index in ECs (B), and intravascular leukocytes (C), in fatal CM; using Spearman’s rank correlation test.

The percentage of neurons that expressed NF-κB p65 correlated positively with total score for histopathological changes (Spearman’s rank correlation, *r*_*s*_ = 0.678; *p* = 0.045) (Figure 10A). There was a significant positive correlation between nuclear ECs NF-κB index and ECs apoptotic index (*r*_*s*_ = 0.717; *p* = 0.030) (Figure 10B), and between intravascular leukocyte NF-κB index and leukocyte apoptotic index in the CM group (*r*_*s*_ = 0.696; *p* = 0.037) (Figure 10C). However, no association was established between NF-κB p65 and caspase-3 expression in neurons, glial cells, and the following clinical parameters: age, haemoglobin, WBC, time to death, admission, and last parasite count.

## Discussion

### NF-κB p65 and apoptosis in cerebral malaria

The expression of NF-κB p65 and the occurrence of apoptosis was investigated in the brains of patients who had died from CM, hoping to determine correlations between NF-κB p65 cell signaling events and apoptosis in various cellular components of the brain--neurons, glial cells, ECs, and intravascular leukocytes. Nuclear NF-κB p65 positive cells indicate the activation of NF-κB p65. NF-κB p65 activation in these cell types as well as the presence of activated glial cells in the damaged brain (areas of ring hemorrhage and Durck’s granuloma) and associated blood vessels suggests that histopathological changes in CM is mediated by the NF-κB p65 signaling pathway. NF-κB responded variably in different cell types as a consequence of malaria infection. Glial cells are known to be sensitive and respond quickly to pathogens and injury. Among the glial cells observed, oligodendrocytes were considered more sensitive to NF-κB p65 activation than astrocytes (glial cell differentiation based on morphology). Purkinje neurons are more resistant to NF-κB p65 activation than other neurons. The presence of glial cells exhibiting NF-κB p65 activation close to the blood vessels may be an immune response secondary to the leakage of soluble mediators from the disruption of vascular permeability in CM.

The activation of NF-κB p65 expression and the induction of the apoptosis process in the neurons and glial cells in CM imply important cell signaling processes. In *P. falciparum* malaria, the process of PRBC-EC cytoadhesion leads to impairment of the BBB, which subsequently allows passage of soluble mediators released by malaria parasites and the host immune response to the brain parenchyma [20]. These soluble factors may trigger the activation of NF-κB in various cellular components of the brain. The results demonstrated that activation of NF-κB and apoptosis occurred in neurons, glial cells, ECs and vascular leukocytes of fatal CM however, both neurons and glial cells showed no correlation between NF-κB p65 expression and cleaved caspase-3, suggesting that the NF-κB p65 signaling pathway is not the main culprit for neuronal and glial cell apoptosis in CM. It is possible that other cell signaling events and stimuli from soluble mediators during the process of malaria infection may contribute to neuronal and glial cell apoptosis. On the contrary, ECs and intravascular leukocytes showed positive correlations between NF-κB p65 activation and cleaved caspase-3 expression suggesting that NF-κB p65 activation modulates apoptosis in ECs and intravascular leukocytes in CM. Previous *in vivo* studies by Tripathi *et al.*, demonstrated that PRBC exposure induced nuclear translocation of NF-κB in brain ECs and was associated with intercellular adhesion molecule 1 (ICAM-1) expression [3,4]. At the transcript level, apoptotic genes were among the members of up-regulated genes [4]. The study suggests that alteration in expression of apoptotic genes does not translate to functional apoptosis probably due to the balancing effect of positive and negative signaling regulators of apoptosis which determines whether apoptosis will occur or not [4]. The association between NF-κB signaling process and the occurrence of apoptosis at the molecular level still needs to be clarified. In this study however, the linkage between NF-κB signaling process and the apoptotic changes in the brain ECs and vascular ECs of CM is documented. Apoptosis of ECs has been reported to be caused by several mechanisms during malaria infection, including (1) sequestration of PRBCs, which is associated with the overexpression of ICAM-1 receptor on ECs surface [21-23] and highly toxic derivative molecules [24], which contribute to apoptosis by caspase-8 and 9 dependence [8], (2) pro-inflammatory cytokines secreted by activated leukocytes [25], and macrophages, such as TNF [26-28], which may act as apoptotic ligands bound to the death receptors, leading to the induction of apoptotic pathways, (3) malaria products, such as glycosylphosphatidylinositol (GPIs) of *P. falciparum* inducing nitric oxide (NO) and oxidative stress [29], and (4) a role of perforin-mediated cytosis by cytotoxic T lymphocyte, leading to apoptosis of ECs [24,30]. These mechanisms, together with the NF-κB signaling process in the present study, could contribute to the cause of apoptosis in ECs in CM, leading to BBB dysfunctions. In intravascular leukocytes, NF-κB activation has a positive correlation with apoptosis, a finding contributing to the role of NF-κB cell signaling induced apoptosis in CM. This finding may reflect the relationship of NF-κB p65 function with the regulation of apoptotic genes. In addition, the expression of NF-κB p65 in the intravascular leukocyte in the CM brain tissue is consistent with a previous report that demonstrated that the activation of NF-κB p65 in the circulating peripheral blood mononuclear cells (PBMCs) of malaria patients could be due the suppression effect of high levels of interleukin (IL) -10 [31].

## Conclusions

The activation of NF-κB p65 and apoptosis was established in the neurons, glial cells, ECs and intravascular leukocytes of patients who died from CM. This study demonstrates that NF-κB is one of the signaling molecules that modulates apoptosis in the brain ECs and intravascular leukocytes of fatal cerebral malaria. This represents a significant contribution to the understanding of the progression of human cerebral malaria.

## Competing interests

The authors declare that they have no competing interests.

## Authors’ contributions

CP and KN carried out the histopathology and immunohistochemistry work, preliminary data analysis and wrote the first draft of the manuscript. YM and UC assisted in the study design and manuscript edits. PV formulated the research idea and designed the experiments, gave laboratory and technical support, supervised, and revised the final manuscript. All authors read and approved the final manuscript.

## References

[B1] Berendt AR Ferguson DJ Gardner J Turner G Rowe A McCormick C Roberts D Craig A Pinches R Elford BC Molecular mechanisms of sequestration in malaria Parasitology 1994 108 Suppl S19 28 8084651 10.1017/s0031182000075685

[B2] Clark IA Rockett KA The cytokine theory of human cerebral malaria Parasitol Today 1994 10 410 412 10.1016/0169-4758(94)90237-2 15275552

[B3] Tripathi AK Sullivan DJ Stins MF *Plasmodium falciparum*-infected erythrocytes increase intercellular adhesion molecule 1 expression on brain endothelium through NF-kappaB Infect Immun 2006 74 3262 3270 10.1128/IAI.01625-05 16714553 PMC1479273

[B4] Tripathi AK Sha W Shulaev V Stins MF Sullivan DJ Jr *Plasmodium falciparum* infected erythrocytes induce NF-kappa B regulated inflammatory pathways in human cerebral endothelium Blood 2009 114 4243 4252 10.1182/blood-2009-06-226415 19713460 PMC2925626

[B5] Tripathi T Aggarwal A NF-kappa B transcription factor: a key player in the generation of immune response Curr Sci India 2006 90 519 531

[B6] Thompson CB Apoptosis in the pathogenesis and treatment of disease Science 1995 267 1456 1462 10.1126/science.7878464 7878464

[B7] Krajewska M Wang HG Krajewski S Zapata JM Shabaik A Gascoyne R Reed JC Immunohistochemical analysis of in vivo patterns of expression of CPP32 (Caspase-3), a cell death protease Cancer Res 1997 57 1605 1613 9108467

[B8] Pino P Vouldoukis I Kolb JP Mahmoudi N Desportes-Livage I Bricaire F Danis M Dugas B Mazier D *Plasmodium falciparum*-infected erythrocyte adhesion induces caspase activation and apoptosis in human endothelial cells J Infect Dis 2003 187 1283 1290 10.1086/373992 12696008

[B9] Hemmer CJ Lehr HA Westphal K Unverricht M Kratzius M Reisinger EC *Plasmodium falciparum* Malaria: reduction of endothelial cell apoptosis *in vitro* Infect Immun 2005 73 1764 1770 10.1128/IAI.73.3.1764-1770.2005 15731077 PMC1064913

[B10] Wassmer SC de Souza JB Frere C Candal FJ Juhan-Vague I Grau GE TGF-beta1 released from activated platelets can induce TNF-stimulated human brain endothelium apoptosis: a new mechanism for microvascular lesion during cerebral malaria J Immunol 2006 176 1180 1184 16394007 10.4049/jimmunol.176.2.1180

[B11] Sanni LA The role of cerebral oedema in the pathogenesis of cerebral malaria Redox Rep 2001 6 137 142 10.1179/135100001101536238 11523587

[B12] Medana IM Chan-Ling T Hunt NH Redistribution and degeneration of retinal astrocytes in experimental murine cerebral malaria: relationship to disruption of the blood-retinal barrier Glia 1996 16 51 64 10.1002/(SICI)1098-1136(199601)16:1<51::AID-GLIA6>3.0.CO;2-E 8787773

[B13] Medana IM Hunt NH Chaudhri G Tumor necrosis factor-alpha expression in the brain during fatal murine cerebral malaria: evidence for production by microglia and astrocytes Am J Pathol 1997 150 1473 1486 9095002 PMC1858172

[B14] Deininger MH Kremsner PG Meyermann R Schluesener H Macrophages/microglial cells in patients with cerebral malaria Eur Cytokine Netw 2002 13 173 185 12101073

[B15] Deininger MH Meyermann R Trautmann K Morgalla M Duffner F Grote EH Wickboldt J Schluesener HJ Cyclooxygenase (COX)-1 expressing macrophages/microglial cells and COX-2 expressing astrocytes accumulate during oligodendroglioma progression Brain Res 2000 885 111 116 10.1016/S0006-8993(00)02978-4 11121536

[B16] Medana IM Day NP Hien TT Mai NT Bethell D Phu NH Farrar J Esiri MM White NJ Turner GD Axonal injury in cerebral malaria Am J Pathol 2002 160 655 666 10.1016/S0002-9440(10)64885-7 11839586 PMC1850649

[B17] Konstantinidou AE Givalos N Gakiopoulou H Korkolopoulou P Kotsiakis X Boviatsis E Agrogiannis G Mahera H Patsouris E Caspase-3 immunohistochemical expression is a marker of apoptosis, increased grade and early recurrence in intracranial meningiomas Apoptosis 2007 12 695 705 10.1007/s10495-006-0001-4 17143787

[B18] Charafe-Jauffret E Tarpin C Bardou VJ Bertucci F Ginestier C Braud AC Puig B Geneix J Hassoun J Birnbaum D Jacquemier J Viens P Immunophenotypic analysis of inflammatory breast cancers: identification of an 'inflammatory signature' J Pathol 2004 202 265 273 10.1002/path.1515 14991891

[B19] Ghosh S Karin M Missing pieces in the NF-kappaB puzzle Cell 2002 109 Suppl S81 96 11983155 10.1016/s0092-8674(02)00703-1

[B20] Lackner P Burger C Pfaller K Heussler V Helbok R Morandell M Broessner G Tannich E Schmutzhard E Beer R Apoptosis in experimental cerebral malaria: spatial profile of cleaved caspase-3 and ultrastructural alterations in different disease stages Neuropathol Appl Neurobiol 2007 33 560 571 17442059 10.1111/j.1365-2990.2007.00833.x

[B21] Porta J Carota A Pizzolato GP Wildi E Widmer MC Margairaz C Grau GE Immunopathological changes in human cerebral malaria Clin Neuropathol 1993 12 142 146 8100753

[B22] Silamut K Phu NH Whitty C Turner GD Louwrier K Mai NT Simpson JA Hien TT White NJ A quantitative analysis of the microvascular sequestration of malaria parasites in the human brain Am J Pathol 1999 155 395 410 10.1016/S0002-9440(10)65136-X 10433933 PMC1866852

[B23] Turner GD Morrison H Jones M Davis TM Looareesuwan S Buley ID Gatter KC Newbold CI Pukritayakamee S Nagachinta B White NJ Berendt AR An immunohistochemical study of the pathology of fatal malaria: evidence for widespread endothelial activation and a potential role for intercellular adhesion molecule-1 in cerebral sequestration Am J Pathol 1994 145 1057 1069 7526692 PMC1887431

[B24] Pino P Taoufiq Z Nitcheu J Vouldoukis I Mazier D Blood–brain barrier breakdown during cerebral malaria: suicide or murder? Thromb Haemost 2005 94 336 340 16113823 10.1160/TH05-05-0354

[B25] Meager A Cytokine regulation of cellular adhesion molecule expression in inflammation Cytokine Growth Factor Rev 1999 10 27 39 10.1016/S1359-6101(98)00024-0 10379910

[B26] Bienvenu AL Gonzalez-Rey E Picot S Apoptosis induced by parasitic diseases Parasit Vectors 2010 3 106 10.1186/1756-3305-3-106 21083888 PMC2995786

[B27] Brown H Hien TT Day N Mai NT Chuong LV Chau TT Loc PP Phu NH Bethell D Farrar J Gatter K White N Turner G Evidence of blood–brain barrier dysfunction in human cerebral malaria Neuropathol Appl Neurobiol 1999 25 331 340 10.1046/j.1365-2990.1999.00188.x 10476050

[B28] Medana IM Hunt NH Chan-Ling T Early activation of microglia in the pathogenesis of fatal murine cerebral malaria Glia 1997 19 91 103 10.1002/(SICI)1098-1136(199702)19:2<91::AID-GLIA1>3.0.CO;2-C 9034826

[B29] Schofield L Novakovic S Gerold P Schwarz RT McConville MJ Tachado SD Glycosylphosphatidylinositol toxin of Plasmodium up-regulates intercellular adhesion molecule-1, vascular cell adhesion molecule-1, and E-selectin expression in vascular endothelial cells and increases leukocyte and parasite cytoadherence via tyrosine kinase-dependent signal transduction J Immunol 1996 156 1886 1896 8596041

[B30] Potter S Chan-Ling T Ball HJ Mansour H Mitchell A Maluish L Hunt NH Perforin mediated apoptosis of cerebral microvascular endothelial cells during experimental cerebral malaria Int J Parasitol 2006 36 485 496 10.1016/j.ijpara.2005.12.005 16500656

[B31] Punsawad C Krudsood S Maneerat Y Chaisri U Tangpukdee N Pongponratn E Nantavisai K Udomsangpetch R Viriyavejakul P Activation of nuclear factor kappa B in peripheral blood mononuclear cells from malaria patients Malar J 2012 11 191 10.1186/1475-2875-11-191 22682094 PMC3422190

